# Synovial sarcoma presenting as an intra-articular mass in a pediatric patient: a case report

**DOI:** 10.1186/s12891-020-03312-3

**Published:** 2020-05-07

**Authors:** Omar A. Al-Mohrej, Saeed A. Al-Jarallah, Hamad H. Al-Dakhil Allah, Rajeev Pant, Zayed S. Al-Zayed

**Affiliations:** Department of Orthopaedics, King Faisal Specialist Hospital and Research Centre, Riyadh, Saudi Arabia

**Keywords:** Synovial sarcoma, Pediatrics, Misdiagnosis, Case report

## Abstract

**Background:**

Synovial sarcoma (SS) is one of the reported sarcomas in the pediatric and adult populations. Delay in diagnosis and treatment is common in SS cases. SS may be excised before the correct diagnosis is made.

**Case presentation:**

we present a case involving a 4-year-old boy who visited our service with complaints of left knee pain and limited knee flexion. Initially, the child was diagnosed with osteochondromatosis. Surgical excision was opted, and initial histopathological examination revealed a fibrous histiocytoma. The slide and blocks were then brought to the King Faisal Specialist Hospital Research Center (KFSH&RC) and histopathologic analysis has shown a well-circumscribed nodule in the synovium with a sub-synovial monomorphic spindle cell sarcoma, confirmed by fluorescence in situ hybridization (FISH).

**Conclusions:**

Therefore, we strongly recommend considering all differential diagnoses for soft-tissue masses when planning surgical management.

## Background

Synovial sarcoma (SS) is one of the sarcomas which reported in the pediatric and adult populations [1]. The peak incidence is within the third decade of life [2]. SS accounts for 8–15% of all soft-tissue sarcoma (STS) cases [3, 4]. SS is the most common nonrhabdomyosarcoma STS (NRMS-STS) with an incidence rate of 0.5 to 0.7/1,000,000 in the pediatric population [3, 5, 6]. Moreover, 30% of SS cases are noted in patients aged ≤20 years [6].

Although it can occur anywhere in the body, SS commonly arises in soft tissues adjacent to large joints of the upper and lower extremities [7]. The symptoms vary, and SS patients present with a painful palpable mass, which grows slowly; it takes sometime before patients present with discernible symptoms, resulting in a delay in diagnosis [3, 8]. Thus, SS is a malignancy with poor prognosis due to high risk of local invasiveness and a propensity to metastasize [9].

Radiological examinations, such as plain X-ray, computed tomography (CT), and magnetic resonance imaging (MRI) are the “first-line” examinations used to evaluate SS. However, SS is definitively diagnosed by histological examination of a biopsy sample.

Despite its name, SS does not develop from synovial tissue [10]. SS was so named due to the resemblance between SS cells and primitive synoviocytes. The origin of SS is unclear. The (X;18)(p11;q11) translocation results in fusion of the homologous gene at Xp11 (SSX1, SSX2, or SSX4) and the SYT gene on chromosome 18. Two fusion proteins (SYT-SSX1 and SYT-SSX2) function as either proto-oncogene activators or tumor suppressor gene inhibitors [11]. SS18 rearrangement is a recognized aberration in SS [12].

There is a strong relationship between the histologic subtype of the tumor and either of these two fusion proteins. The majority of the SYT-SSX2 tumors present a monophasic phenotype involving only a spindle cell component [11], while almost all biphasic tumors, containing both epithelial and spindle cell components, express a SYT-SSX1 transcript [11].

The treatment of SS depends on several factors. Surgical excision is the mainstay of treatment. We present a case of SS in a 4-year-old child who was initially misdiagnosed. The case adheres to CARE guidelines [13].

## Case presentation

The patient’s parents were informed that data concerning the case would be submitted for publication, and they provided consent for the same. A 4-year-old boy with global developmental delay and bronchial asthma presented to a private clinic with complaints of left knee pain and limited knee flexion. This pain was irregularly experienced on movement. He mainly showed localized tenderness over the anterolateral aspect of the left knee.

**Fig. 1:**
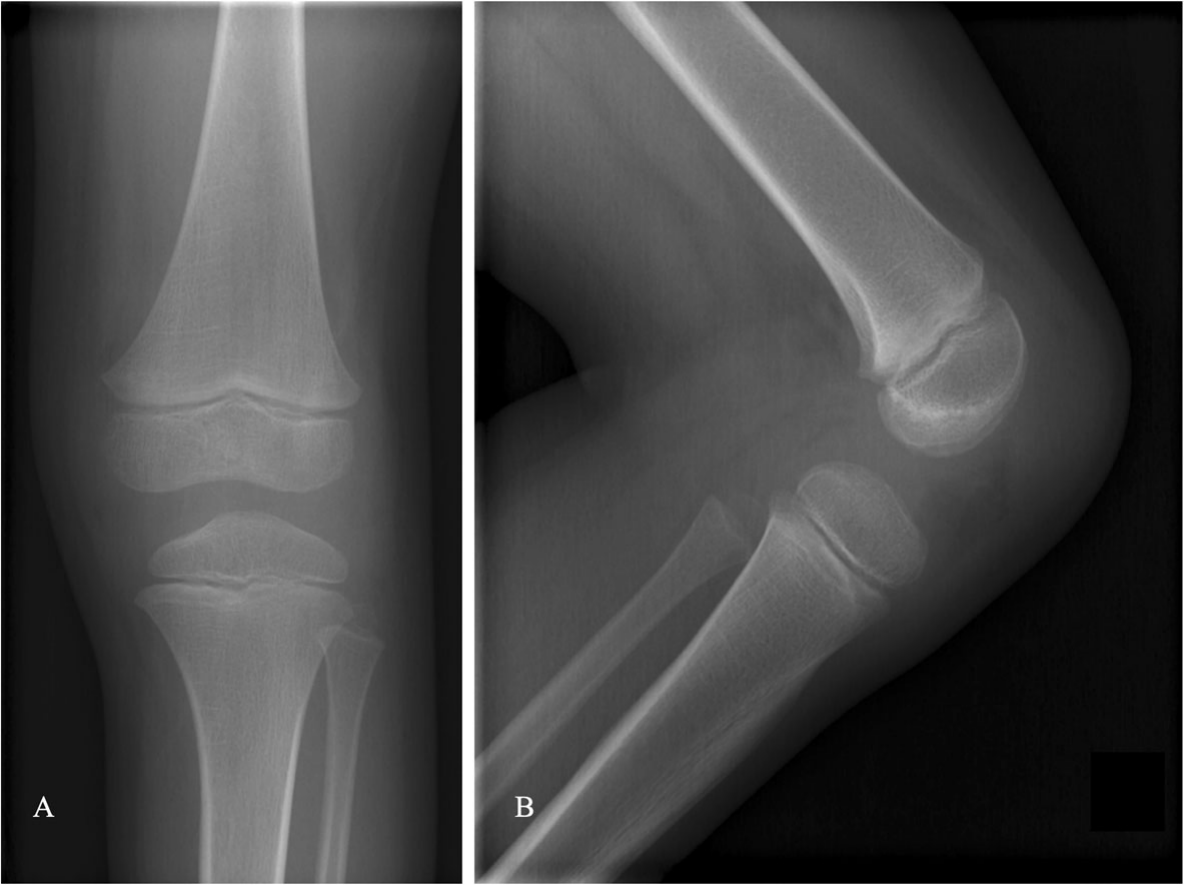
Plain radiograph of the concerned knee showing non-specific findings

**Fig. 2:**
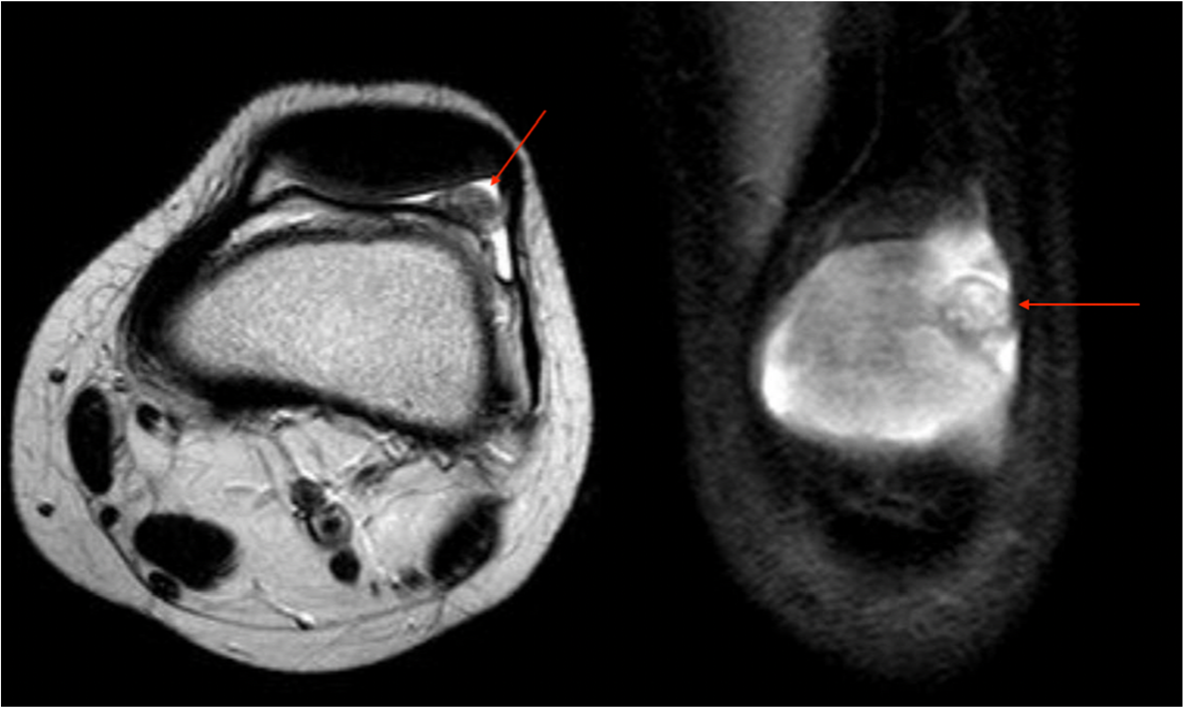
Axial T2 and coronal PD fat sat MRI scans of the left knee showing the lesion (red rows). Unfortunately, limited quality without gadolinium enhancement of the left knee as outside MR study were submitted as pre-op MRI. As well, no gradient cartilage sequences were included in the study. So, we were unable to comment about the above-mentioned sequences

Initial knee radiographs revealed no remarkable findings [Fig. 1]. Computed tomography (CT) and bone scans were also unremarkable. However, magnetic resonance imaging (MRI) revealed the possible presence of a focal small osteocartilaginous lesion of the left knee [Fig. 2].

**Fig. 3:**
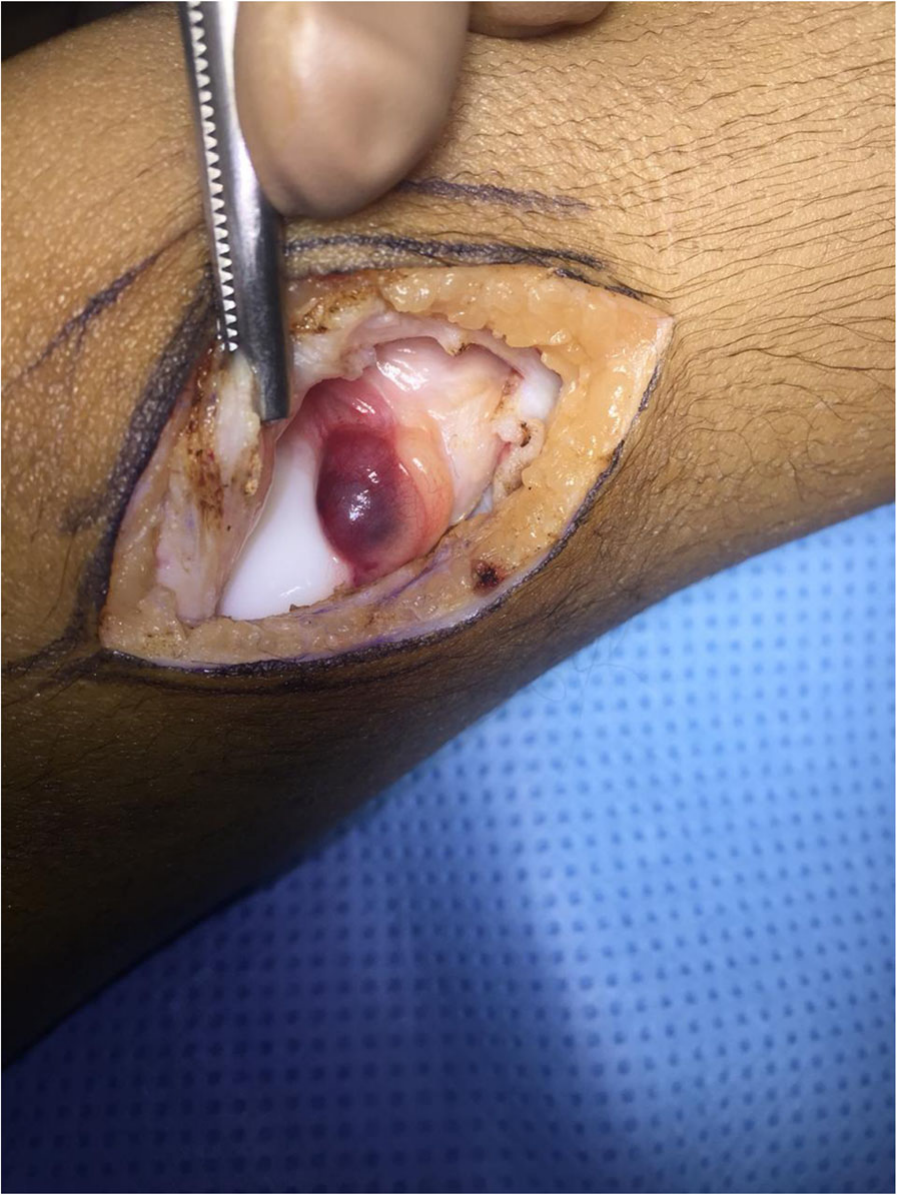
Intra-operative photograph showing a pedunculated 2 × 2 Cm small lesion, which appeared like a blood clot

After discussion with the family, we opted for surgical excision through a longitudinal incision. We observed a pedunculated 2 × 2 cm small lesion resembling a blood clot arising from the border of the lateral femoral condyle [Fig. 3]; the lesion was completely excised, and the condyle was flushed together with the removal of the synovium around it. The excised sample was sent for histopathological examination.

**Fig. 4:**
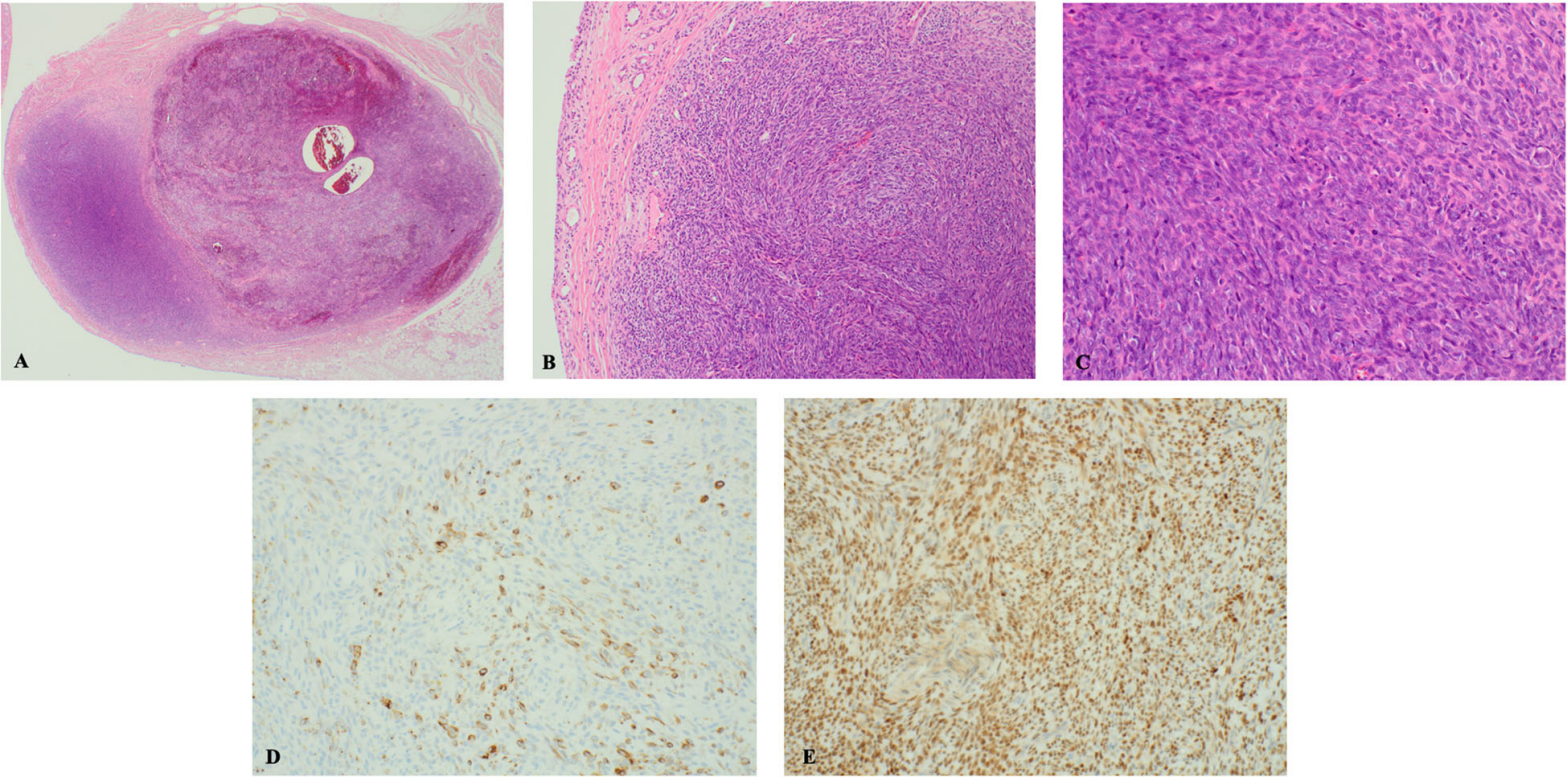
**a** Well-circumscribed nodule in the synovium (H&E staining, 2×) **b** Sub-synovial monomorphic spindle cell sarcoma (synovial lining is on the left side); (H&E staining, 10×). **c** Monomorphic spindle cell sarcoma with brisk mitotic activity (H&E stain, 20×). **d** Tumor cells showing patchy cytoplasmic positivity for cytokeratin 19 (immunohistochemistry CK19, 20×) **e**. Tumor cells showing diffuse nuclear positivity for TLE-1 (immunohistochemistry TLE-1, 20×). H&E: hematoxylin and eosin

The pathologist at that clinic thought that the lesion was benign and labeled it as a fibrous histiocytoma. He had performed limited staining; therefore, we brought the slide and blocks to the King Faisal Specialist Hospital Research Center (KFSH&RC). While the lesion was reported to be 2 × 2 cm in size, the whole resected surgical specimen measured 2 × 1.5 × 0.7 cm. Histopathology revealed a well-circumscribed nodule in the synovium with sub-synovial monomorphic spindle cell sarcoma with brisk mitotic activity in the hematoxylin and eosin stained sections. The tumor cells showed patchy cytoplasmic positivity for cytokeratin 19 and diffuse nuclear positivity for transducin-like enhancer of split 1 (TLE-1; Fig. 4]. Fluorescence in situ hybridization (FISH) revealed positivity for SS18(SYT)(18q11.2) in 68% of the interphase nuclei.

This case was reviewed by a multidisciplinary team involving medical and radiation oncologists to establish the optimum care protocols for the child. Radiation was deferred due to the long-term risks of radiation in young children, which include growth disturbance and secondary malignancy.

The pediatric oncology team was involved due to the possibility that chemotherapy may be required. However, the pediatric oncology team preferred not to administer chemotherapy since the tumor was small and completely excised; we agreed with their decision.

The patient was monitored regularly. During the immediate post-operative examination, he was lying comfortably with no tenderness anywhere. The wound healed nicely; there was no erythema or discharge. The patient showed full range of motion for the concerned knee. As well, the patient was examined in his last visit to us, 1-year post operatively, with similar examination findings.

Staging of the tumor was performed with positron emission tomography-computed tomography (PET-CT), and non-contrast CT of the chest was performed. No evidence of metastatic disease was observed. Increased abnormal fluorodeoxyglucose (FDG) uptake was noticed at the site of the known primary tumor, indicating postoperative inflammatory change.

The patient was evaluated at the sarcoma clinic at 2 and 6 weeks, and at 3 months thereafter. Clinical, radiological, and laboratory evaluations were performed at each follow-up. The patient was followed-up for a year with no evidence of local recurrence or metastatic disease on surveillance MRI and CT scans of the chest, abdomen, and pelvis. These were done 3 times, and the final PET-CT scan was with no significant FDG avid residual/recurrent or metastatic disease. So, our plan was not to do unless there are clinical symptoms.

## Discussion and conclusion

Delay in diagnosis and treatment is common in SS cases [6]. SS may be excised before the correct diagnosis is made [14]. Therefore, it is not unexpected that a significant number of studies reported excision of lumps that were later identified as SS [14–16]. According to Chandrasekar et al., referrals after an excision due to incorrect diagnoses account for 19–53% of the patients who visit specialized centers [14].

Since the synovial membrane covers a large part of the knee, the intra-articular lesions of the knee are common. Lesions in the knee joint are commonly benign and arise from inflammatory or degenerative articular disease [17]. However, soft tissue tumors should always be considered since SS is observed in 10% of the cases [17].

Here, we present an unfortunate scenario where a mesenchymal tumor that was potentially aggressive was incorrectly diagnosed as a benign tumor preoperatively. This pitfall is a great learning point for those who make daily decisions regarding surgical treatment. Such lesions should be diagnosed systematically. The location of the lesion must be ascertained, whether it is intra or extra-articular. The anatomical structure involved must then be defined.

Synovial osteochondromatosis, pigmented villonodular synovitis (PVNS), or synovial hemangiomas could be differential diagnoses for tumor-like lesions in the knee joint. While MRI plays a major role in the evaluation and description of intra-articular lesions of the knee, distinguishing intra-articular synovial sarcomas from benign tumors is challenging since no specific radiological features of SS have been established [18]. SS is hypointense on T1-weighted MRI images and hyperintense on T2-weighted MRI images. It may appear multilobulated with well-defined borders. Occasionally, internal septa are observed [17]. However, SS tends to be larger, shows small effusion volumes, and may be calcified [18]. The patients are generally younger and of male sex as reported by Nordemar et al [18]. As well, SS has very low Apparent diffusion coefficient (ADC) values which may help differentiation from hemangioma (high ADC) but may be not from PVNS. So, it is important to know the role of gradient recalled echo (GRE) imaging for PVNS differentiation and Diffusion-weighted imaging (DWI) [19, 20]. Unfortunately, limited quality MRI without gadolinium enhancement, gradient cartilage and GRE sequences were done pre-op in the private center.

Moreover, the other main concern in this report was the incorrect diagnosis by the pathologist in the private center. However, the incorrect diagnosis had not affected our final management since complete resection with clear margins remains the treatment of choice for localized SS in children [21]. Although SS was not initially suspected, we performed wide excision with negative margins. However, it is important to determine whether arthroscopic resection is contraindicated in such situations. We think there are no absolute guidelines in cases where the diagnosis is unclear. Two cases similar to ours were treated with arthroscopic resection; both patients have recurrent disease [22, 23]. Therefore, it is better to not perform knee arthroscopy when in doubt to avoid catastrophic joint contamination with sarcoma.

Although the use of adjuvant radiotherapy and/or chemotherapy was suggested for children who undergo surgery [24], the oncologists in our center decided not to subject the patient to such therapies due to his age and because his skeleton was not well-developed. The decision was also attributable to the negative surgical margins. It is important to mention that there are no randomized trials evaluating the use of chemotherapy for SS [6].

Tumor stage, grade, size and male sex have adverse prognostic significance in SS, and the impact must be confirmed in prospective studies [6]. However, SS prognosis and the related factors remain a controversial topic. The tendency for recurrence and metastases is well-established. However, studies on pediatric patients aged less than 5 years are rare. The size of the tumor in our patient correlates with the findings of previous studies that reported a good prognosis. Additionally, the quality of the surgical excision directly affects tumor control.

Our report emphasizes the importance of teamwork since the patient was misdiagnosed initially although appropriate diagnostic evaluations were performed. Therefore, we strongly recommend considering all differential diagnoses for soft-tissue masses when planning surgical management. Therefore, selection of proper diagnostic modalities, careful assessment of biopsy samples, and adequate surgical excision are essential for SS treatment.

## Data Availability

Not applicable.

## Abbreviations

SS: Synovial sarcoma
STS: Soft tissue sarcoma
CT: Computed tomography
MRI: Magnetic resonance imaging
ADC: Apparent diffusion coefficient
GRE: Gradient recalled echo
DWI: Diffusion-weighted imaging
FISH: Fluorescence in situ hybridization
